# Decreased diastolic wall strain is associated with adverse left ventricular remodeling even in patients with normal left ventricular diastolic function

**DOI:** 10.1007/s12574-014-0238-9

**Published:** 2014-12-16

**Authors:** Min-Kyung Kang, Sungbae Ju, Hee-Sun Mun, Seonghoon Choi, Jung Rae Cho, Namho Lee

**Affiliations:** Cardiology Division, Kangnam Sacred Heart Hospital, Hallym University Medical Center, Seoul, South Korea

**Keywords:** Diastolic wall strain, Left ventricular, Diastole, Echocardiography

## Abstract

**Background:**

The pathophysiology of diastolic dysfunction is complex, but can be simply described as impaired LV myocardial relaxation and/or increased LV stiffness. The objective of this study is to clarify true normal left ventricular (LV) diastolic function and early stage of diastolic dysfunction before relaxation abnormality develops in patients with normal LV diastolic function using simple diastolic wall strain (DWS) in South Korea.

**Methods:**

DWS which is a non-invasive, load-independent, and reproducible estimator of LV stiffness using two-dimensional echocardiography using the difference between posterior wall thickness in systole and diastole to approximate LV stiffness. A total of 349 consecutive patients with normal LV diastolic function by echocardiography were enrolled. According to DWS, patients were divided into two groups: high DWS (≥median 175) vs. low DWS (<median 174).

**Results:**

Patients with low DWS were more obese and showed higher blood pressure, and had more prevalent hypertension and hyperlipidemia. In addition, those with low DWS had higher LV end-systolic volume, LV mass index, E/E’ and lower ejection fraction and E’ velocity. Among them, higher LVESV and LVMI were independently associated with low DWS.

**Conclusions:**

These data suggests that simple DWS might be helpful in identifying a subgroup of subtle diastolic dysfunction. Our data suggest that early change of diastolic dysfunction might start with abnormal LV geographic changes preceding functional changes.

## Introduction

Left ventricular (LV) diastolic dysfunction is common in the general population, and is associated with incident heart failure and increased mortality [1, 2]. The pathophysiology of diastolic dysfunction is complex, but can be simply described as impaired LV myocardial relaxation and/or increased LV stiffness, both of which can lead to increased LV filling pressures at rest or with exercise [3]. In contrast to asymptomatic diastolic dysfunction, sometimes we encounter unexpected normal LV diastolic patterns in older patients concomitant with hypertension, diabetes, or coronary artery disease. However, even if they met the criteria for normal LV diastolic function, their LV diastolic function might not be the same as true normal LV diastolic function of young healthy subjects. Recently, a non-invasive, load-independent, and reproducible estimator of LV stiffness using M-mode echocardiography, namely diastolic wall strain (DWS), has been proposed [4, 5]. DWS, an extension of linear elastic theory, uses the difference between posterior wall thickness in systole (PWTs) and diastole (PWTd) to approximate LV stiffness, which decreased wall thinning during diastole reflects reduced LV compliance and distensibility, and thus, increased LV stiffness [4]. DWS correlated well with the diastolic stiffness constant measured invasively in an animal model [4]. Clinically, DWS is also useful in assessing diastolic stiffness, and more advanced diastolic stiffness is associated with worse outcomes in heart failure with preserved ejection fraction (HFpEF) [5]. Recently, Takagi et al. [6] reported that low DWS is associated with raised post-exercise E/E’ ratio in elderly patients without obvious myocardial ischemia and patients with low DWS are likely to develop raised E/E’ after exercise. Therefore, the objective of this study was to determine the relationship between DWS and cardiac structure and function, and to see whether increased diastolic stiffness as assessed by DWS is a predictive value for subtle diastolic dysfunction or clinical implications even in patients with normal LV diastolic function. If there were any differences, it might be helpful to distinguish subtle diastolic dysfunction in patients who have predisposing factors for diastolic dysfunction from true normal diastolic function.

## Methods

### Study design and participants

We conducted an observational cross-sectional study in which we enrolled 349 patients who met the criteria for normal LV diastolic function among 6,277 subjects who underwent transthoracic echocardiography at Kangnam Sacred Heart Hospital between April 2012 to May 2013 [40 ± 12 years, 153 (44 %) women]. We enrolled patients who met criteria for normal LV diastolic function as both E/A, E’/A’ ratio were 1.1 or higher, deceleration time (DT) was 142–220 ms, and septal E’ velocity was 10 cm/s or higher [7], and patients with overt heart diseases [severe valvular diseases, systolic heart failure (HF), or pericardial diseases], LV ejection fraction (EF) ≤50 %, E/E’ ≥15, or age ≥80 were excluded from the study. On 140 patients, carotid ultrasound was performed, and among them, 72 patients underwent also brachial-ankle pulse wave velocity (baPWV). We also collected participant data on demographic, anthropometric, and inflammatory parameters.

### Transthoracic echocardiography (TTE)

TTE was performed using standard techniques with a 2.5-MHz transducer. The standard 2-D and Doppler echocardiography was performed using a commercially available echocardiographic machine (Vivid 7R GE Medical System, Horten, Norway). LV end-diastolic dimensions (LVEDD), end-diastolic interventricular septal thickness (IVSTd), and end-diastolic left ventricular posterior wall thickness (PWTd) were measured at end-diastole according to the standards established by the American Society of Echocardiography [8]. LV EF was determined by the biplane Simpson’s method [9]. Maximal LA volume was calculated using the Simpson method [10] and indexed to the body surface area (LA volume index; LAVI). Left ventricular mass (LVM) was calculated using the Devereux formula [11]: LVM = 1.04[(LVEDD + IVSTd + PWTd)^3^ − (LVEDD)^3^] − 13.6. Thereafter, the LV mass index (LVMI) was calculated and indexed to body surface area, and LV hypertrophy was defined by an LV mass index >95 g/m^2^ in women or >115 g/m^2^ in men. Calculation of relative wall thickness (RWT) by the formula 2× (PWTd)/LVEDD permits categorization of an increase in LV mass as either concentric (RWT >0.42) or eccentric (RWT ≤0.42) hypertrophy and allows identification of concentric remodeling (normal LV mass with increased RWT) [8]. Volumes were obtained using biplane Simpson’s rule from the apical 4- and 2-chamber views. The endocardial border was manually traced by an experienced sonographer according to the recommendations of the American Society of Echocardiography, leaving the papillary muscles and trabeculations within the cavity [12]. Measurements of LV end-diastolic volume (LVEDV), LV end-systolic volume (LVESV), and EF were obtained off-line, with LVEDV measurements at the frame just prior to mitral valve closure and LVESV measured on the image with the smallest LV cavity. Additionally in the apical 4-chamber view the ventricular length was measured in end-diastole, from the plane of the annulus to the apex. DWS was calculated as [(PWTs) − (PWTd)/(PWTs)] using M-mode echocardiography [4, 5].

Mitral flow velocities were recorded in the apical four-chamber view. Mitral inflow measurements included the E/A ratio and the peak early (E) and peak late (A) flow velocities. The tissue Doppler of the mitral annulus movement was also obtained from the apical four-chamber view. A 1.5-mm sample volume was placed sequentially at the lateral and septal annular sites. The analysis was performed for early diastolic (E’) and late diastolic (A’) peak tissue velocities. As a noninvasive parameter for LV stiffness, the LV filling index (E/E’) was calculated by the ratio of transmitral flow velocity to annular velocity. Adequate mitral and tissue Doppler image (TDI) signals were recorded in all patients [13].

### Carotid ultrasound

A high-resolution B-mode ultrasound (Vivid 7R GE Medical Systems, Horten, Norway) equipped with a 7.5-MHz linear array transducer was used for carotid ultrasonography. In the longitudinal view, carotid intima-media thickness (IMT) was determined as the distance from the media adventitia interface to the intima lumen interface on the far wall in a region free of plaque [14]. The examiner assessed the presence of carotid plaques, which were defined as focal structures that encroached into the lumen by at least 100 % of the surrounding IMT value. Common carotid artery IMT (CCA-IMT) was measured between the origin of the carotid bulb and a point 10 mm proximal to the CCA, and the carotid bulb IMT (CB-IMT) was measured in the carotid bulb region. CCA-IMT and CB-IMT values were determined as the average of the maximum IMT of the left and right CCA and CB.

### Pulse wave velocity (PWV)

PWV was measured using a VP-2000 automated device (Colin Co., Komaki, Japan). The details of this device and the measurement method have been described elsewhere [15]. Right and left brachial-ankle PWV (baPWV) were measured simultaneously. The subjects were permitted to rest in a supine position for 15 min prior to the measurements. The pressure waveforms of the brachial and tibial arteries were obtained from the occlusion and monitoring cuffs wrapped around the upper arms and lower legs. Measurements were performed in a quiet-controlled room (22 ± 1 °C), with the subjects in the overnight fasted state. The baseline brachial systolic blood pressure (SBP) and diastolic blood pressure (DBP), pulse rate (PR), and baPWV were measured simultaneously.

### Statistical analysis

Data are expressed as numbers and percentages for categorical variables, and mean ± standard deviation for continuous variables. Correlations between continuous variables and DWS were examined using Spearman’s correlation analysis. Patients were assigned based on the median DWS to either the low or the high (< or ≥ median DWS) group. Two groups were compared using Fisher’s exact test or Mann–Whitney’s *U* test. Independent contributing factors to the DWS were investigated by multiple logistic regression analysis of significant variables in the univariate analysis. Variables that strongly influenced others were excluded from the multivariate analysis. Male and female were compared using each analysis. A *p* value <0.05 was considered significant. All data were analyzed using StatView software (SPSS for Macintosh, version 10.0.7a, SPSS, Inc., Chicago, IL., USA) was performed.

## Results

### Baseline demographic characteristics

The median (25th to 75th percentiles) DWS was 0.36 (0.33–0.42) of the overall study population. In the previous adult study (mean age 56.7 ± 8.3 years), the normal value of the DWS was reported to be 0.40 ± 0.07 [4], and another recent study showed DWS was 0.39 ± 0.06 in the adult group aged 30–50 years [16]. Our median value was slightly lowered compared to previous normal cut-off values. We divided study populations into two groups according to the DWS, and Table 1 shows the characteristics of the study population with higher (>median) or lower (≤median) DWS. Patients with lower DWS were more obese and had a higher prevalence of hypertension and hyperlipidemia when compared with patients with higher DWS. The use of angiotensin-converting enzyme inhibitors or angiotensin receptor blocker among anti-hypertensive medications was more common in patients with lower DWS. Systolic and diastolic BP was higher in patients with lower DWS.

**Table 1:** Baseline demographic characteristics

Variables	Higher DWS (*n* = 175)	Lower DWS (*n* = 174)	*p*
Age (years)	39 ± 12	41 ± 12	0.107
Women	79 (52 %)	74 (48 %)	0.667
BMI (kg/m^2^)	23.2 ± 4.1	24.1 ± 3.9	0.050
SBP (mmHg)	116 ± 14	121 ± 17	0.001
DBP (mmHg)	70 ± 9	74 ± 11	<0.001
Pulse rate (beats/min)	67 ± 11	66 ± 11	0.619
Hypertension	26 (15 %)	39 (22 %)	0.075
Diabetes	20 (11 %)	24 (14 %)	0.523
Hyperlipidemia	18 (11 %)	33 (19 %)	0.033
Current medication			
Aspirin	16 (9 %)	23 (13 %)	0.308
CCB	15 (9 %)	24 (14 %)	0.173
β-blockers	8 (5 %)	14 (8 %)	0.270
ACEis or ARBs	21 (12 %)	38 (22 %)	0.025
Diuretics	5 (3 %)	7 (4 %)	0.770
Current smokers	43 (25 %)	43 (25 %)	1.000

*BMI* body mass index, *SBP* systolic blood pressure, *DBP* diastolic BP, *CCB* calcium channel blocker, *ACE* angiotensin-converting enzyme, ARB angiotensin II receptor blocker

### Echocardiographic parameters

**Fig. 1:**
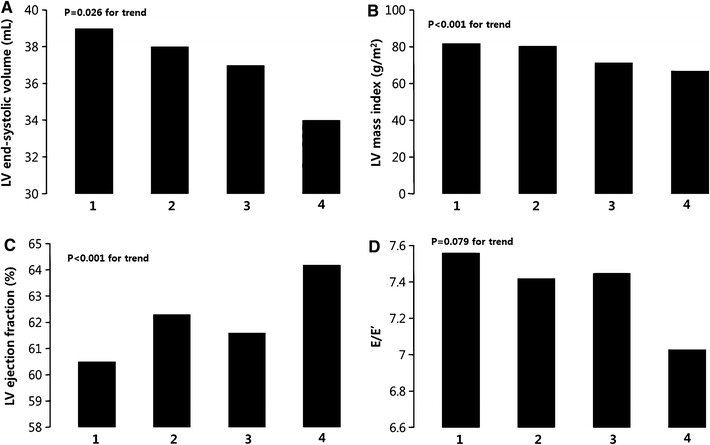
Echocardiographic parameters versus quartiles of diastolic wall strain. **a** Left ventricular end-systolic volume, **b** left ventricular mass index, **c** ejection fraction, **d** E/E’

Table 2 shows echocardiographic parameters of the study population. Figure 1 shows the relationship between DWS quartiles and echocardiographic LV volume and mass and systolic and diastolic indices. We compared differences of structural parameters and functional parameters in patients with normal LV diastolic function according to DWS.

**Table 2:** Baseline echocardiographic characteristics

Variables	Higher DWS (*n* = 175)	Lower DWS (*n* = 174)	*p*
LAVI (ml/m^2^)	21.6 ± 5.8	22.7 ± 6.4	0.074
LVEDD (mm)	50.2 ± 3.5	50.5 ± 4.0	0.476
LVESD (mm)	32.2 ± 3.1	33.4 ± 4.0	0.001
LVEDV (ml)	128.2 ± 27.1	131.0 ± 30.8	0.372
LVESV (ml)	34.2 ± 10.1	38.5 ± 12.4	<0.001
LV mass index (g/m^2^)	69.2 ± 14.0	81.23 ± 17.8	<0.001
IVSTd (mm)	7.1 ± 1.2	7.9 ± 1.3	<0.001
IVSTs (mm)	11.0 ± 1.7	11.9 ± 1.7	<0.001
PWTd (mm)	7.0 ± 1.1	8.3 ± 1.2	<0.001
PWTs (mm)	12.4 ± 1.7	11.9 ± 1.7	0.001
Relative wall thickness	0.28 ± 0.04	0.32 ± 0.04	<0.001
LV remodeling			
Normal	174 (99 %)	157 (90 %)	0.002
Concentric remodeling	0 (0 %)	2 (1 %)	
Eccentric LVH	1 (0.6 %)	14 (8 %)	
Concentric LVH	0 (0 %)	1 (0.3 %)	
LV ejection fraction (%)	63.7 ± 5.3	61.3 ± 5.2	<0.001
E (cm/s)	77.6 ± 14.9	77.2 ± 13.0	0.798
A (cm/s)	51.8 ± 11.6	53.0 ± 12.7	0.354
E/A ratio	1.56 ± 0.43	1.53 ± 0.46	0.495
Deceleration time (ms)	162.9 ± 13.8	166.1 ± 12.6	0.023
IVRT (ms)	82 ± 15	82 ± 18	0.660
Septal E’ (cm/s)	13.8 ± 3.6	12.5 ± 3.1	0.007
Septal A’ (cm/s)	7.6 ± 1.4	7.4 ± 1.4	0.323
Septal E’/A’ ratio	1.52 ± 0.43	1.47 ± 0.38	0.175
Septal E/E’ ratio	6.1 ± 1.3	6.5 ± 1.5	0.016
Septal S’ (cm/s)	7.8 ± 1.4	7.6 ± 1.4	0.097

*LAVI* left atrial volume index, *LVEDD* left ventricular end-diastolic dimension, *LVESD* LV end-systolic dimension, *IVST* interventricular septal wall thickness, *PWT* posterior wall thickness, *d* diastole, *s* systole, *IVRT* isovolumic relaxation time

#### Diastolic wall strain and LV structural parameters

**Fig. 2:**
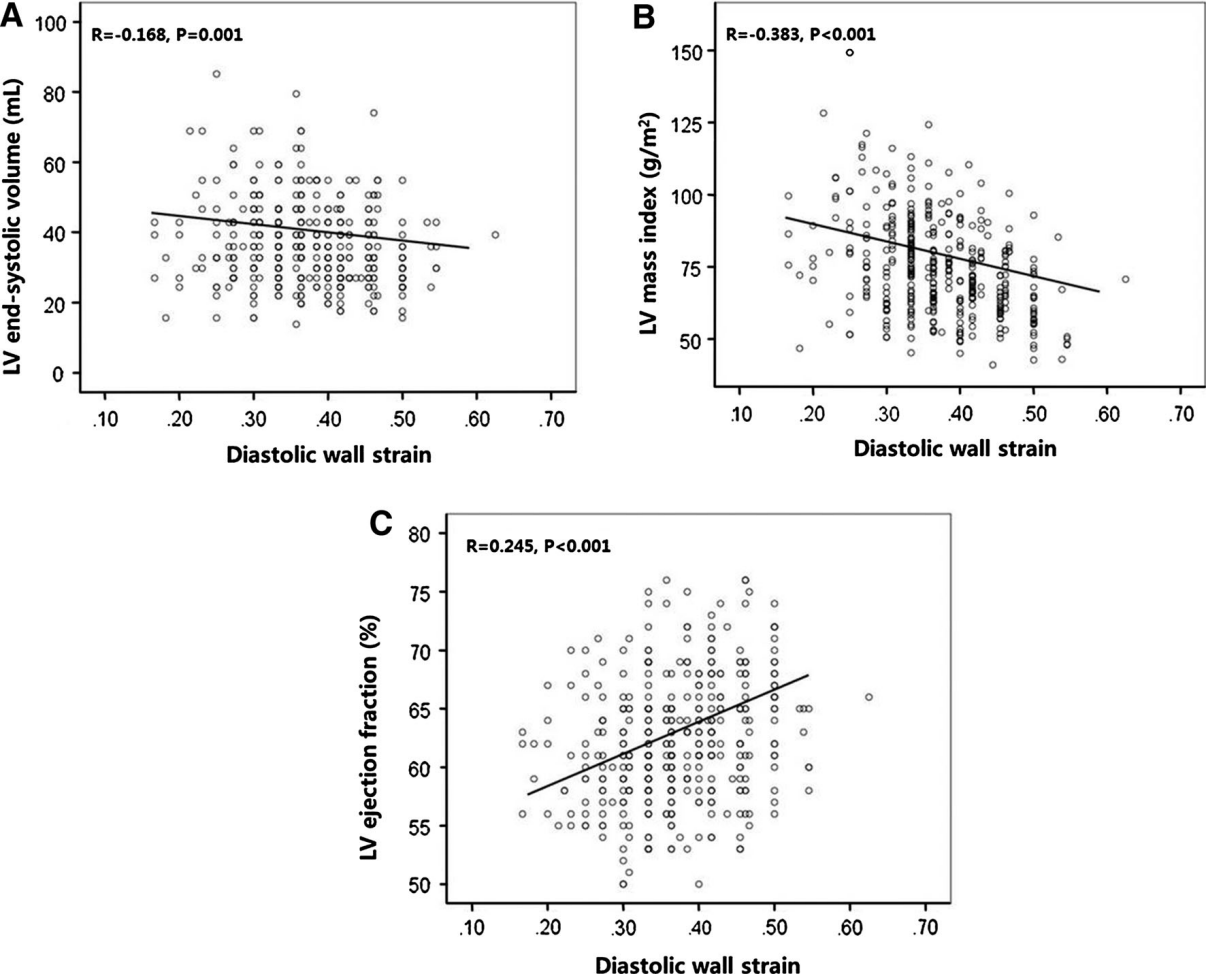
Scatterplots depicting the relationship between DWS and echocardiographic parameters. **a** Left ventricular end-systolic volume, **b** left ventricular mass index, **c** ejection fraction

When compared with patients with higher DWS, patients with lower DWS showed larger LV end-systolic dimension and end-systolic volume. In addition, LV mass index was much higher in patients with lower DWS. In terms of adverse LV remodeling, patients with lower DWS had more abnormal geometry. DWS was inversely correlated with LVESV (*r* = −0.168, *p* = 0.001) and LVMI (*r* = −0.383, *p* < 0.001). Scatterplots depicting the relationship between DWS and LVESV and LVMI are shown in Fig. 2a and b, respectively.

#### Diastolic wall strain and LV functional parameters

LV EF was slightly but significantly lower in patients with lower DWS (EF: 63.7 ± 5.3 vs. 61.3 ± 5.2 %, *p* < 0.001). In addition, E’ velocity also slightly but significantly lower (E’ 11.2 ± 2.2 vs. 10.5 ± 1.8 cm/s, *p* = 0.002) and E/E’ ratio was higher (E/E’ 7.1 ± 1.3 vs. 7.5 ± 1.5, *p* = 0.016) in patients with lower DWS among indices reflective of diastolic function. Figure 1c and d shows the relationship between DWS quartiles and EF and E/E’, respectively, and Fig. 2c shows scatterplots depicting the relationship between DWS and LV EF.

### Pulse wave velocity and carotid ultrasound

Among the whole population, on 140 patients carotid ultrasound was performed and on 72 patients baPWV was performed, and the results are shown in Table 3. There were no statistically significant differences between the two groups. However, baPWV was higher in patients with low DWS (PWV 13.1 ± 17.1 vs. 14.2 ± 28.7 m/s, *p* = 0.064) with marginal statistical significance. Carotid IMT was also slightly increased in patients with lower DWS.

**Table 3:** Parameters of carotid ultrasonography and PWV

Variables	Higher DWS	Lower DWS	*p*
IMT (mm)	0.55 ± 0.11	0.59 ± 1.6	0.090
Maximal plaque (mm)	1.92 ± 0.61	1.95 ± 0.70	0.903
Presence of plaque	23 (33 %)	31 (44 %)	0.224
PWV (m/s)	13.1 ± 17.1	14.2 ± 28.7	0.064
Central SBP (mmHg)	121 ± 15	128 ± 21	0.122
Central DBP (mmHg)	74 ± 7	78 ± 13	0.230
AI	73 ± 17	75 ± 16	0.636
AI75 %	71 ± 15	71 ± 14	0.983

*IMT* intima-medial thickness, *PWV* pulse wave velocity, *SBP* systolic blood pressure, *DBP* diastolic BP, *AI* augmentation index

### Correlations of continuous variables with DWS

As shown in Table 4, BMI, BP, LV ESD, ESV, LVMI, RWT, EF, DT, E’ velocity, E/E’ ratio and S’ velocity were significantly correlated with DWS, whereas age and LAVI did not. Echocardiographic findings showed that variables associated with LV geometry (LV ESD, ESV, LVMI, and RWT) were closely correlated with DWS. Of the tissue Doppler parameters, DT, E’ velocity, E/E’ ratio, and S’ velocity were correlated with DWS.

**Table 4:** Correlation of clinical and echocardiographic variables with DWS

	*ρ*	*p*
Age	−0.067	0.182
Body mass index	−0.125	0.013
Systolic blood pressure	−0.166	0.001
Diastolic blood pressure	−0.175	0.001
Hyperlipidemia	−0.060	0.239
Left ventricular (LV) end-diastolic dimension	−0.052	0.305
LV end-systolic dimension	−0.120	0.018
LV end-diastolic volume	−0.042	0.409
LV end-systolic volume	−0.171	0.001
LV mass index	−0.313	<0.001
Relative wall thickness	−0.386	<0.001
LV ejection fraction	0.116	0.022
Left atrial volume index	−0.056	0.272
E velocity	0.014	0.781
A velocity	0.011	0.822
E/A ratio	0.041	0.413
Deceleration time	−0.164	0.001
E’ velocity	0.165	0.001
A’ velocity	0.067	0.185
E’/A’ ratio	0.065	0.198
E/E’ ratio	−0.106	0.036
S’ velocity	0.122	0.016
Pulse wave velocity	−0.160	0.158
Carotid intima-media thickness	−0.164	0.040

**Table 5:** Multiple logistic regression analysis to predict factors associated with lower diastolic wall strain

Variables	Odds ratio	95 % confidence interval	*p*
BMI	1.035	0.957–1.119	0.393
SBP	0.991	0.967–1.015	0.467
DBP	1.035	0.998–1.073	0.084
Hyperlipidemia	2.628	0.898–0.695	0.078
LVESD	0.997	0.682–1.459	0.997
LVESV	1.122	1.071–1.166	<0.001
LVMI	1.091	1.064–1.120	<0.001
EF	0.759	0.696–0.829	<0.001
DT	1.016	0.979–1.055	0.392
E’	0.998	0.864–1.153	0.979
E/E’	1.166	0.948–1.435	0.147
S’	1.445	0.957–2.181	0.109
Carotid IMT	0.507	0.015–17.748	0.708

*BMI* body mass index, *SBP* systolic blood pressure, *DBP* diastolic BP, *LVESD* left ventricular end-systolic dimension, *ESV* end-systolic volume, *LVMI* LV mass index, *EF* ejection fraction, *DT* deceleration time

### Factors associated with lower diastolic wall strain

Higher BMI, elevated systolic and diastolic BP, increased LV ESV and LVMI, lower EF and E’, and higher E/E’ were parameters found related to lower DWS via univariate analysis. Among them, increased LVESV (OR 1.122, CI 1.071–1.166, *p* < 0.001) and LVMI (OR 1.091, CI 1.064–1.120, *p* < 0.001) and lower EF (OR 0.759, CI 0.696–0.829, *p* < 0.001) were independently associated with lower DWS in patients with normal LV diastolic function.

## Discussion

We found that patients with lower DWS were more obese, had higher BP, and more prevalent hypertension and hyperlipidemia. Echocardiographic parameters showed that patients with lower DWS had larger LVESD, LVESV, and LVMI as a result more prevalent eccentric LVH. In addition, LV EF was slightly but significantly lowered in patients with lower DWS, and E’ velocity was lower and E/E’ ratio was higher in patients with lower DWS. Among those parameters, LVESV, LVMI, and EF were independently associated with decreased DWS in patients with normal LV diastolic function according to our study. Among few patients performed carotid ultrasound and baPWV, carotid IMT was slightly increased and baPWV was also higher with marginal statistical significance.

Ohtani et al. [5] found that decreased diastolic wall stress was associated with adverse remodeling and poor outcomes in HFpEF. They reported that patients with DWS median ≤ (0.33) had higher LVMI, RWT, E/E’, Doppler-estimated LV end-diastolic pressure to LV end-diastolic volume ratio, LAVI, and brain natriuretic peptide (BNP) levels than those with DWS > median. In our study, higher BP and more prevalent hypertension and hyperlipidemia were found in patients with low DWS (<0.36). Those factors are risk factors for HFpEF [17] except hyperlipidemia, which is rather more related to HF with reduced EF in terms of myocardial infarction. In addition, Ohtani et al. [5] also compared the median value of DWS patients with HFpEF (0.33 ± 0.08) with controls (0.40 ± 0.07). In our study, the median value was 0.36, which was slightly lowered compared to previous studies, i.e., 0.40 from Ohtani et al. and 0.39 from Suzue et al. [17].

According to our study, patients with lower DWS showed not only enlarged cardiac size but also diminished indices for both systolic and diastolic function. For starters, LVESD, LVESV, and LVMI were all larger in those with lower DWS. Therefore, eccentric LVH was more prevalent in those patients compared to patients with higher DWS. A previous study by Ohtani et al. [5] showed among HFpEF patients, those with lower DWS showed more enlarged LVESD, LVMI with more abnormal LV geometry, but showed similar LVEDD, LVEDV and septal wall thickness like ours. In terms of LV function, patients with lower DWS showed slightly but significantly decreased EF compared to patients with higher DWS in our study. Even there was a significant difference exist in LV EF, both EF were within normal ranges (EF 63.7 ± 5.3 vs. 61.3 ± 5.2 %, *p* < 0.001). We assume that this difference was mainly due to larger LVESV in patients with lower DWS. DWS was also correlated with some diastolic indices (E’ velocity, E/E’ ratio also slightly but significantly) like previous studies [3, 5].

We also performed carotid ultrasound in 157 patients and baPWV in 79 patients. To mention conclusion first, there were no statistically significant differences between the two groups. However, patients with lower DWS had slightly increased mean carotid IMT and increased baPWV with marginal statistical significance (PWV 13.1 ± 17.1 vs. 14.2 ± 28.7 m/s, *p* = 0.064). PWV is generally accepted as the most simple, non-invasive, and validated indicator of arterial stiffness [18]. It is also well known that increased arterial stiffness is an early marker of systemic atherosclerosis and it also demonstrates an independent predictive value for cardiovascular events in patients with hypertension [19, 20], diabetes [21], end-stage renal disease [22], in elderly subjects [23], and the general population [24]. Furthermore, baPWV value 14.2 ± 28.7 m/s for patients with lower DWS in our study is very close to the upper normal limits for median age 40 years [25], which might be a marker for subclinical or an early stage of atherosclerosis in conjunction with higher prevalence of hypertension, hyperlipidemia, and increased carotid IMT. However, we performed baPWV in only a small number of patients and also our data did not have clinical outcomes, those findings failed to be translated to clinical implications. Therefore, further larger-scale prospective trials are needed to determine the relationship of DWS and PWV in clinical aspects.

## Strengths and limitations of the study

This study was, to the best of our knowledge, the first study to see early change of diastolic dysfunction among patients with normal LV diastolic function using DWS. Our study showed that lower DWS is associated with larger LVESV, LVMI with slightly decreased LV EF even in patients with normal LV diastolic function. In addition, those who had lower DWS were more obese and had more prevalent hypertension and hyperlipidemia. Therefore, our data suggests that adverse cardiac remodeling might occur before overt diastolic dysfunction occurs. Limitations include the lack of clinical outcomes. As mentioned earlier, further larger-scale prospective trials are needed to determine the relationship of DWS and clinical aspects in patients without overt heart disease. In addition, the lack of invasive gold standard measurement of diastolic stiffness should be mentioned for limitations.

## Conclusions

Our study shows that patients with lower DWS had larger LVESD, LVESV, and LVMI as a result of more prevalent eccentric LVH. In addition, LV EF was slightly but significantly lowered in patients with lower DWS, and E’ velocity was lower and E/E’ ratio was higher in patients with lower DWS. Among those parameters, increased LVESV, LVMI, and decreased EF were independently associated with decreased DWS in patients with normal LV diastolic function. Our data suggests that DWS might be helpful in detecting subtle diastolic dysfunction in patients with normal LV diastolic function, and this change starts with LV geometrical remodeling before functional changes.
